# Identification of candidate host-specificity genes in *Exserohilum turcicum* using comparative genomics and transcriptomics

**DOI:** 10.1093/g3journal/jkaf084

**Published:** 2025-04-11

**Authors:** Mara J Krone, Pragya Adhikari, Pummi Singh, Tiffany M Jamann, Santiago X Mideros

**Affiliations:** Department of Crop Sciences, University of Illinois Urbana-Champaign, Urbana, IL 61801, USA; Department of Crop Sciences, University of Illinois Urbana-Champaign, Urbana, IL 61801, USA; Department of Crop Sciences, University of Illinois Urbana-Champaign, Urbana, IL 61801, USA; Department of Crop Sciences, University of Illinois Urbana-Champaign, Urbana, IL 61801, USA; Department of Crop Sciences, University of Illinois Urbana-Champaign, Urbana, IL 61801, USA

**Keywords:** whole-genome sequence, dothideomycete, host specificity, RNA-seq, effectors, secondary metabolites, homologs, genome assembly

## Abstract

*Exserohilum turcicum* causes northern corn leaf blight and sorghum leaf blight. While the same species cause disease in both crops, the strains are host-specific. Here, we report the sequence and de novo annotated assemblies of one sorghum- and one maize-specific *E. turcicum* strain. The strains were sequenced using the PacBio Sequel II system. The total genome length for both assemblies was between 44 and 45 Mb with N50 of ∼2.5 Mb. Ninety-eight percent of the Benchmarking Universal Single-Copy Orthologs (BUSCO) for both assemblies had complete status. The estimated number of genes was 11,762 and 12,029 in the sorghum- and maize-specific isolates, respectively. Funannotate, EffectorP, SignalP, and transcriptome data were used to create functional annotation of each genome. The whole-genome comparison identified ten large-scale inversions and three translocations between the maize- and sorghum-specific strains, along with homologous genes and gene duplications. RNA was sequenced from the maize- and sorghum-specific isolate 10 days post-inoculation in maize and sorghum and from axenic cultures. Gene expression data from *planta* and axenic growth experiments were compared for each strain. Candidate host-specificity genes were identified by combining results from whole-genome comparison, synteny analysis, gene annotations, and transcriptome data. Overall, this study identified several candidate host-specificity genes that provide insights into *E. turcicum* interaction with its hosts.

## Introduction

*Exserohilum turcicum* (syn. *Setosphaeria turcica*) (Dothideomycetes; Ascomycota) is a pathogen of maize (*Zea mays*) and sorghum (*Sorghum bicolor*). In maize, *E. turcicum* causes northern corn leaf blight (NCLB), a disease of global importance (Savary *et al*. 2019). NCLB consistently ranks among the top 10 most destructive diseases of maize in the United States and Ontario (Mueller *et al*. 2020, 2024). In sorghum, *E. turcicum* causes sorghum leaf blight (SLB) that can result in significant yield losses to both forage and grain sorghum. SLB is one of the most important and widespread sorghum diseases in tropical and subtropical regions (Das and Rajendrakumar 2016).

*E. turcicum* strains are host-specific, meaning a strain can only infect either maize or sorghum (Hamid and Aragaki 1975). Strains of *E. turcicum* have been classified as *formae speciales zeae,* for strains that infect maize, and f. sp. *sorghi,* for strains that infect sorghum. Population genetics studies suggest *E. turcicum* has coevolved with maize in Mexico (Borchardt *et al*. 1998; Ferguson and Carson 2004). *E. turcicum* populations collected from maize and sorghum in South Africa showed significant levels of genetic differentiation (Nieuwoudt *et al*. 2018). *E. turcicum* strains with contrasting host specificity can mate and produce viable offspring in a laboratory setting (Hamid and Aragaki 1975). We previously reported the identification of quantitative trait loci (QTL) for virulence to maize, using a biparental population created by a cross between a sorghum- and a maize-specific strain (Singh *et al*. 2022). Other studies have suggested the presence of two distinct loci associated with virulence on maize and sorghum (Hamid and Aragaki 1975; Singh *et al*. 2022). However, the specific genes underlying host specificity in *E. turcicum* remain unknown.

One approach to identify genes conferring host specificity in pathogens is to use comparative genomics combined with transcriptomic data (Bates *et al*. 2024). Previous studies have identified host-specificity genes which prevent infection in some host plants, often referred as Avirulence genes (Avr). Several Avr genes that confer host specificity have been identified in *Magnaporthe oryzae* (Sordariomycetes; Ascomycota), a pathogen that infects >50 grass species, but strains are host-specific similarly to *E. turcicum* (Inoue 2017; Li *et al*. 2020). For example, the Avr gene *PWL2* in *M. oryzae* prevents infection of weeping love grass (Sweigard *et al*. 1995) and the Avr genes *PWT3* and *PWT4* are present in strains that infect oats but prevent infection in wheat (Inoue 2017). The majority of *M. oryzae* Avr genes encode small-secreted proteins, most likely effectors, which can be identified from genome sequences (Sperschneider and Dodds 2022).

Secondary metabolites have also been identified to play a key role in host specificity by preventing infection in certain hosts. Polyketide synthase (PKS) and nonribosomal peptide synthetase (NRPS) genes comprise two related classes of megasynthases and are responsible for the biosynthesis of secondary metabolites (Nivina *et al*. 2019). Hybrids of these enzyme classes are called either PKS–NRPS or NRPS–PKS. In *M. oryzae*, the gene *ACE1* encodes a putative hybrid PKS–NRPS enzyme, which is recognized by the Pi33 resistance gene in rice leading to immunity (Bohnert *et al*. 2004). A similar example can be found in *E. turcicum*, where the candidate *AvrHt1* gene encodes for a hybrid PKS–NRPS enzyme (Mideros *et al*. 2018). These hybrid genes are responsible for producing bioactive chimeric compounds, such as mycotoxins, which can influence host specificity (Theobald *et al*. 2019). The genes that code for PKS, NRPS, and PKS–NRPS hybrid secondary metabolites can also be identified by genome annotations, contributing to our understanding of the molecular basis of host specificity. Both effectors and secondary metabolites have been identified as key determinants of host-specificity in several pathosystems (Li *et al*. 2020).

In addition to genetic factors that prevent infection, there are also genetic factors that are required for infection in specific hosts, known as virulence genes. These virulence genes can encode proteinaceous host-specific toxins, or host-selective toxins in the case of secondary metabolites (Li *et al*. 2020). Two common examples of the latter are found in *Cochliobolus heterostrophus* and *C. carbonum* (Dothideomycetes; Ascomycota), two species closely related to *E. turcicum*, where the virulence loci are required for host specificity. In these species, the virulence loci were found to be composed of multiple genes and disrupted by chromosomal translocations (Ahn and Walton 1996; Kodama *et al*. 1999). Genome structural variants, such as translocations are now recognized as important regions harboring virulence and pathogenicity genes in fungi (Hartmann 2022). Fungal plant pathogen's genome assemblies generated using long-read sequencing technologies facilitate the identification of such structural variants, enabling the discovery of genomic regions involved in host specificity and infection.

Understanding the genetic architecture underlying *E. turcicum* ability to cause disease on maize and sorghum can help develop novel, durable, and long-lasting forms of resistance. The overall aim of this study was to identify candidate genes responsible for host specificity in *E. turcicum*. The specific objectives were to (i) assemble and annotate the genomes of one sorghum-specific and one maize-specific *E. turcicum* strain, (ii) conduct a whole-genome comparison between these strains, (iii) compare the gene expression profiles of both strains, and (iv) leverage these genome and transcriptome comparisons to identify candidate host-specificity genes.

## Materials and methods

### Fungal strains and sequencing

The maize-specific isolate, Et52B, was previously used as a parental strain for two fungal populations (Mideros *et al*. 2018; Singh *et al*. 2022). The sorghum-specific isolate, 15St008, was collected from Champaign County, Illinois in 2015 (Zhang *et al*. 2020). High molecular weight DNA was extracted using the NucleoBond HMW DNA Extraction kit (Macherey-Nagel, Allentown, PA) and submitted for library preparation and sequencing to Roy J. Carver Biotechnology Center at the University of Illinois at Urbana-Champaign. The genomic DNA was sequenced using Pacbio sequel II system (Pacific Biosciences, Menlo Park, CA). The gDNA was sheared using a Megaruptor 3 (Diagenode, Denville, NJ) to an average fragment length of 13 Kb. Sheared gDNA was converted to a library with the SMRTBell Express Template Prep kit 2.0 (Pacific Biosciences, Menlo Park, CA). The pooled library was sequenced on a SMRTCell 8M on a Sequel IIe using the circular consensus sequencing (CCS) mode and a 30-h movie time. CCS analysis was done in an instrument with SMRTLink V10.1.0 to generate HiFi reads.

For the transcriptome data, the maize inbred line B73 was inoculated with Et52B, and the sorghum inbred line BTx623 was inoculated with 15St008. The plants were inoculated as described by Singh *et al*. (2022) and grown under identical environmental conditions at the Plant Care Facility at the University of Illinois Urbana-Champaign. The experiment was set up in a completely randomized design with three replications, where each experimental unit consisted of a pot with an inoculated plant. Inoculations were conducted at the V3 stage (3 leaves present, 21–28 days after planting). The infected *in planta* tissue was flash frozen 10 days after inoculation for RNA extractions. Three replications of the two isolates were also grown as axenic cultures. The cultures were started on lactose-casein hydrolysate agar (LCA) media (Tuite 1969) under 12-h light at 25°C and allowed to grow for 15 days. Three to four plugs of the LCA culture were then transferred to potato dextrose broth (PDB). The PDB cultures were placed on a shaker (120 rpm) at room temperature for 5 days. Mycelia were harvested by vacuum filtration on sterile filter paper placed in a Büchner funnel, rinsed with sterile water, and lyophilized. Lyophilized mycelia was used for RNA extractions.

RNA was extracted using TriZol (Thermo Fisher Scientific, Waltham, MA) following manufacturer instructions and cleaned using an RNA clean and concentrator kit (Zymo Research, Irvine, CA). The RNA quality and integrity were assessed by running the RNA on a 1% agarose gel. A total of 12 RNA samples (three maize, three sorghum, three axenic Et52B, and three axenic 15St008) were submitted for library preparation and sequencing at Roy J. Carver Biotechnology Center at the University of Illinois Urbana-Champaign. The RNA-seq libraries were prepared using a TruSeq Stranded mRNAseq Sample Prep kit (Illumina, San Diego, CA, United States) and quantified using qRT-PCR. The samples were individually barcoded, pooled randomly and sequenced across three lanes for 101 cycles of single-end sequencing on a NovaSeq 6000 (Illumina).

### Genome assembly

HiFi reads were assembled into a genome using hifiasm (Cheng *et al*. 2021). Since the pathogen is haploid, duplication purging was disabled during assembly in hifiasm. The quality of the genomes was assessed using QUAST (Mikheenko *et al*. 2018) and the completeness of the genomes was measured using the benchmarking universal single-copy orthologs software (BUSCO) (Manni *et al*. 2021). The genome-wide repeat families were identified de novo using RepeatModeler (Flynn *et al*. 2020). The known fungal clade repeats (odb10) were obtained from Repbase and classified using Repeat classifier included in the RepeatModeler program. Then, the identified genome-wide repeat families and the known fungal clade repeats were merged in a single file and repeats were masked in the genomes using Repeatmasker.

### Gene prediction and genome annotation

Gene prediction was conducted using the funannotate “predict” pipeline (Palmer and Stajich 2020). Transcriptome data (described below) were used to train the gene prediction models. Functional annotations were completed using the protein sequences output by funannotate “predict” and running InterProScan5 (Jones *et al*. 2014), Eggnog-mapper (Cantalapiedra *et al*. 2021), and antiSMASH (Blin *et al*. 2013). SignalP and Phobius were activated in InterProScan5 to be integrated into the protein function prediction. Final annotations were completed using funannotate “annotate” with the protein-coding models output from the previous annotations to identify Pfam domains, carbohydrate-active enzymes (CAZymes), proteases (MEROPS), and BUSCO groups. The results from InterProScan5, Eggnog-mapper, and antiSMASH were incorporated with funannotate to create the additional annotations of InterPro terms, clusters of orthologous genes (COGs), and gene ontology (GO) terms. The protein amino acid sequences for both genomes were used to predict effectors using EffectorP V 3.0 (Sperschneider and Dodds 2022). The protein sequences that were predicted as effectors by EffectorP V 3.0 were then run through SignalP V 6.0 (Teufel *et al*. 2022) to identify putatively secreted effectors.

### Genome structure comparison

Conserved synteny plots were created to examine the structural variation between the maize-specific strain (Et52B) and the sorghum-specific strain (15St008). The two genomes were aligned using BLAST + (Camacho *et al*. 2009). First, the 15St008 genome was made into a BLAST database using the makeblastdb command. Then, the Et52B genome was aligned to the 15St008 database using the blastn command. A simplified GFF file was created for both genomes that had four columns: species and chromosomes number, gene ID, start position, and stop position. MCScanX (Wang *et al*. 2012) used the output of the BLAST alignment and the simplified GFF file to produce a collinearity file. The collinearity file and the GFF file were then used as input into SynVisio (Bandi and Gutwin 2020) to create the conserved synteny plot. The same methods were used to create conserved synteny plots comparing 15St008 and Et52B individually to the Et28A-V2.0 genome (Ohm *et al*. 2012; Condon *et al*. 2013).

### Genome and gene comparisons

The genome annotations completed with funannotate were compared between the two strains using the funannotate “compare” pipeline with default settings. In addition, OrthoFinder (Emms and Kelly 2019) was used to compare the two strain's genomic sequences and identify genes that have homologs in the other strain, genes that are unique to each strain, and duplicated genes within each strain (paralogs). OrthoFinder recommended practices were followed and amino acid sequences were used as the input for each fungal strain. If a gene had multiple transcripts, only the longest sequence was used as a single representative transcript for the gene.

A phylogenomic analysis was conducted for the polyketide synthases (PKS) and nonribosomal peptide synthetases (NRPS). The PKS–NRPS hybrids were included in both trees. MUSCLE version 3.8.31 (Edgar 2004) was used to align the protein sequences. The phylogenetic analysis was performed using RAxML version 8.2.12 (Stamatakis 2014). Rapid bootstrapping with maximum-likelihood methods was executed using the command raxmlHPC-f a. The parameter -# autoMRE was used to determine the correct number of bootstraps and the parameter -m PROTGAMMAAUTO was used to automatically assess the best model of amino acid substitution.

### Differential gene expression analysis

Initial quality control of the RNA-seq raw reads was performed using FastQC. The Et52B and 15St008 genome assemblies were indexed using STAR v2.7 (Dobin *et al*. 2013) so that they could be used as reference genomes. The reads from the three Et52B inoculated maize samples, and the three Et52B axenic samples were aligned to the indexed maize-specific (Et52B) assembled genome. The reads from the three 15St008 inoculated sorghum samples and the three 15St008 axenic samples were aligned to the indexed sorghum-specific (15St008) assembled genome. The read counts per gene were calculated for each alignment using the subread package featureCounts version 2.0.0 (Liao *et al*. 2014). MultiQC was run on the feature counts for quality control. The multi-mapping and ambiguous reads were removed.

The read counts for each alignment were imported into R version 4.2.1 (R Core Team 2021) for further analysis. The Bioconductor package edgeR was used for quality control and normalization (Robinson *et al*. 2010). The genes with fewer than one count per million (CPM) were filtered out. The trimmed mean of M-values normalization factors was calculated to estimate the most effective library size and to calculate normalized log_2_ CPM values with a prior count of three. This normalized count data were used in the limma-trend analysis to calculate differentially expressed genes (DEGs). The package limma (Ritchie *et al*. 2015) was used to calculate the DEGs between the *in planta* and axenic contrasts for the maize- and sorghum-specific strains. The test statistics were calculated by adjusting the mean variance using the empirical Bayes “shrinkage” method. Genes were considered differentially expressed if the false discovery rate was <0.05.

The DEG data were further investigated by completing a biological process GO term enrichment analysis. TopGO version 2.50.0 (Alexa and Rahnenfuhrer 2022) was used to complete a Kolmogorov–Smirnov (KS) enrichment test with the “elim” algorithm. The KS test used gene scores that were the adjusted *P*-values generated from the DEG analysis. The GO term enrichment analysis was completed for the sorghum- and maize-specific genomes.

### Translation of genetic mapping positions onto the new assemblies

Singh *et al* (2022) mapped virulence to maize to linkage group 4 (scaffold CP054627) and virulence to sorghum to linkage group 1 (scaffold CP054628) using the PacBio genome assembly of strain Et28A (Cao *et al*. 2020) as the reference genome. To translate these positions to the new reference genomes in this study, we identified the sequence for the entire QTL region. For the virulence on maize QTL, we used CP054627.1:1837606-2567647. We then used blastn with default parameters to identify the corresponding contigs on the new maize-strain (ET52B) and the new sorghum-strain (15St008) assemblies. The contig with the lowest *E*-value is reported as the position of the QTL on the new reference assembly.

### Identification of candidate host-specificity genes

The results from the previous genetic mapping and genome structure comparison were used to identify candidate regions for the location of host-specificity genes. Specifically, the maize specificity QTL was mapped to a single contig on the 15St008 genome (see previous section and Results). However, no significant regions were identified for the sorghum-specificity locus through genetic mapping (Singh *et al* 2022). Since structural variants, such as translocations, can distort recombination rates and, therefore, interfere with genetic mapping results (Hartmann 2022), we explored the three translocations between the two strains (see Results) as possible locations for the sorghum-specificity locus. Then, within the identified regions of interest, we focused on genes related to secondary metabolites, including PKS and NRPS genes, CAZymes, and effectors. We analyzed the expression level of these genes during infection of maize and sorghum, as well as their differential gene expression between in planta and axenic samples. We hypothesized that the maize-specificity locus would be expressed in maize but not in sorghum if it was a gene required for infection, or conversely, not expressed in maize but expressed in sorghum if it represented a gene that prevents infection (i.e. an avirulence gene). The same expression pattern was expected for the sorghum-specificity locus (Supplementary Fig. a). Ultimately, a candidate host-specificity gene was identified if it was in one of the regions of interest, classified as a gene of interest and had changes in gene expression.

## Results

### Genome assemblies and their annotations

The Pacbio sequel II system generated 280,372 reads for the maize-specific strain (Et52B) and 463,364 reads for the sorghum-specific strain (15St008). The genome assembly pipeline resulted in 37 contigs for Et52B and 29 contigs for 15St008 (Supplementary Table a). The assembly size for Et52B was 43,984,748 bp with an N50 of 2,450,830 bp, while for 15St008, the assembly size was 45,016,168 bp with an N50 of 2,520,004 bp (Table 1). We found a total of over 98% complete and single-copy BUSCOs for both genomes (Table 1). The percentage of assembly covered by repeats was higher in sorghum-specific isolate (29.11%) when compared with that of maize-specific isolate (19.55%). The estimated number of genes was 12,029 and 11,762 in the maize- and sorghum-specific strains, respectively. In the maize-specific strain, 130 of these genes were tRNA, while in the sorghum-specific strain, 119 genes were tRNA (Table 1). The genome assemblies were deposited at NCBI (accessions: JBMGSY000000000 and JBMGSX000000000, under BioProjects: PRJNA1187605 and PRJNA1187604).

**Table 1.** Comparison of the sorghum- and maize-specific *E. turcicum* genome assemblies.

	Maize-specific strain (Et52B)	Sorghum-specific strain (15St008)
Assembly Size	43,984,748 bp	45,016,168 bp
Largest Contig	4,427,035 bp	3,986,045 bp
Average Contig	1,188,777 bp	1,552,282 bp
Number of Contigs	37	29
Contig N50	2,450,830 bp	2,520,004 bp
Percent GC	50.49%	50.59%
Number of Genes	12,029	11,762
Number of Proteins	12,615	12,340
Number of tRNA	130	119
Complete and single-copy BUSCOs	98.3%	98.2%
Complete and duplicated BUSCOs	0.3%	0.1%
Fragmented BUSCOs	0.3%	0.3%
Missing BUSCOs	1.1%	1.4%
Cytoplasmic effectors	116	104
Apoplastic effectors	248	243

Effectors were predicted using EffectorP and SignalP. A total of 329 effectors were predicted in the maize-specific strain and 317 effectors were predicted in the sorghum-specific strain. EffectorP differentiated between predicted cytoplasmic and apoplastic effectors (Table 1). In both genomes, multiple effectors were predicted by EffectorP to be both cytoplasmic and apoplastic (Supplementary Table b).

A total of 12,745 and 12,459 functional annotations were produced for the maize- and sorghum-specific strain respectively (accessions: JBMGSY000000000 and JBMGSX000000000, under BioProjects: PRJNA1187605 and PRJNA1187604). Protein domains were predicted using both InterProScan and Pfam and compared between strains. Further comparisons of the annotations were conducted using COG and CAZy families. Of the 24 COGs, only three had the same number of genes in both strains (Table 2). Ten COG groups had a higher number of genes in the sorghum-specific strain, while 11 groups had a higher number of genes in the maize-specific strain. Among the six CAZy families, only two had the same number of genes in both strains (Table 2). The sorghum-specific strain had more genes in the auxiliary activity family, whereas the maize-specific strain had more genes in the carbohydrate esterase, glycoside hydrolase, and glycosyltransferase families (Table 2).

**Table 2.** Genome annotation comparison between the sorghum- (15St008) and maize-specific (Et52B) strains. The number of genes annotated in each cluster of orthologous group and carbohydrate-active enzyme family are listed.

	Et52B	15St008
Clusters of orthologous genes		
RNA processing and modification	343	353
Energy production and conversion	388	397
Nucleotide transport and metabolism	114	115
Coenzyme transport and metabolism	191	192
Lipid transport and metabolism	414	420
Translation, ribosomal structure, and biogenesis	418	420
Posttranslational modification, protein turnover, chaperones	622	628
Inorganic ion transport and metabolism	307	310
Intracellular trafficking, secretion, and vesicular transport	506	510
Cytoskeleton	142	146
Cell cycle control, cell division, chromosome partitioning	182	178
Amino acid transport and metabolism	514	508
Carbohydrate transport and metabolism	646	639
Transcription	410	405
Replication, recombination, and repair	423	365
Cell wall/membrane/envelope biogenesis	97	94
Secondary metabolites biosynthesis, transport, and catabolism	573	565
Signal transduction mechanisms	425	422
Defense mechanisms	48	46
Nuclear structure	28	25
Chromatin structure and dynamics	146	146
Cell motility	6	6
Extracellular structures	6	6
Function unknown	2,713	2,682
Carbohydrate-active enzyme families		
Auxiliary activities	119	122
Carbohydrate-binding module	14	14
Carbohydrate esterase	70	68
Glycoside hydrolase	225	222
Glycosyltransferase	78	73
Polysaccharide lyase	15	15
Total CAZymes	521	514

### Genome structure differences

**Fig. 1.**
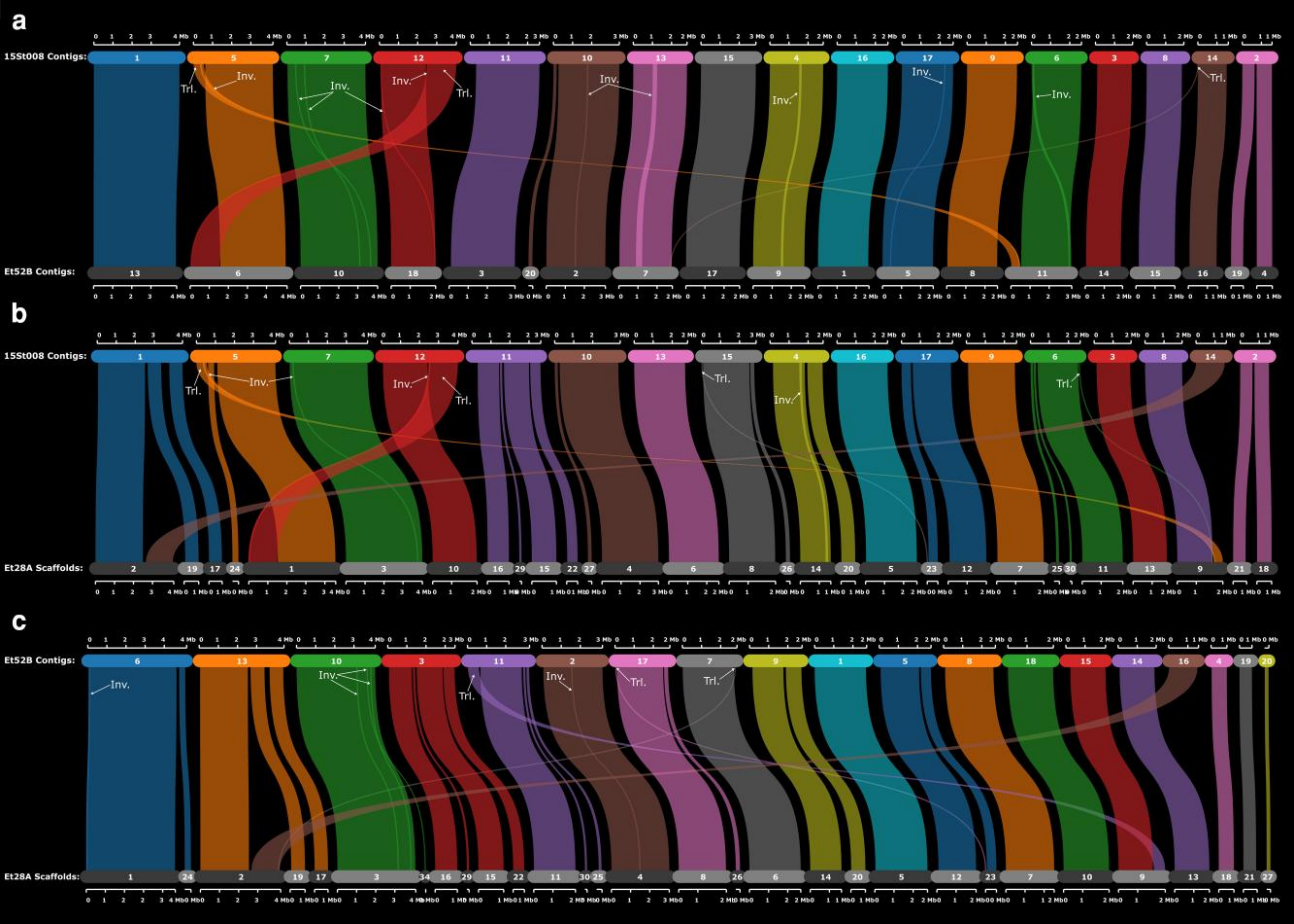
Synteny plots. Inversions between strains are highlighted by a darker color, showing where the inversions are and how large they are. a) Synteny plot between sorghum-specific strain 15St008 and maize-specific strain Et52B. Ten inversions and three translocations were identified between the two strains. b) Synteny plot between sorghum-specific strain 15St008 and maize-specific strain Et28A. Four inversions and two translocations were identified between the two strains. c) Synteny plot between maize-specific strains Et52B and Et28A. Five inversions and three translocations were identified between the two strains. The inversions are highlighted in each synteny plot with a darker color. Inv., inversion; Trl., translocation.

Structural differences were identified from the synteny plots. There were 10 inversions and three translocations between the maize-specific (Et52B) and the sorghum-specific (15St008) genomes (Fig. 1a). Of particular interest was that the fifth contig of the sorghum-specific strain, which was mostly syntenic with the sixth contig of the maize-specific strain, but a small portion of it was syntenic with the eleventh contig of the maize-specific strain. This region of translocation also contained an inversion. Approximately two-thirds of the 15St008 contig 12 was syntenic with Et52B contig 18 (Fig. 1a), while the remaining portion of 15St008 contig 12 was syntenic with the sixth Et52B contig. This region also exhibited an inversion within the translocation.

The synteny analysis between the sorghum-specific strain and the previously available maize-specific strain genome (Et28A-v2.0) (Ohm *et al*. 2012; Condon *et al*. 2013) identified four inversions and four translocations (Fig. 1b). Scaffold 34 of Et28A-v2.0 was not syntenic with any portion of the sorghum-specific strain's genome (not shown in Fig. 1b because of the lack of synteny). The translocations between contigs 5 and 12 of 15St008 and Et52B (Fig. 1a) were also found between 15St008 and Et28A-v2.0 (Fig. 1b). More inversions were found between 15St008 and Et52B (*N* = 10) than between 15St008 and Et28A-v2.0 (*N* = 4), but the four inversions were found in both comparisons. The synteny analysis between the genomes of the two maize-specific strains (Et52B and Et28A-v2.0) identified five inversions and four translocations (Fig. 1c).

### Gene content differences

Gene comparisons with Orthofinder indicated that 5.5% (*N* = 656) of the genes were unique to the maize-specific strain genome (Et52B), while 94.5% (*N* = 11,243) of the Et52B genes had at least one homolog in the sorghum-specific strain genome (15St008). On the other hand, 3.7% (*N* = 435) of the genes were unique to 15St008, while 96.3% (*N* = 11,208) of the 15St008 had at least one homolog in Et52B. There were one-to-one, one-to-many, many-to-one, and many-to-many matches of genes between the two strains for a total of 11,107 homolog groups (Supplementary Table c).

### PKS and NRPS differences

**Fig. 2.**
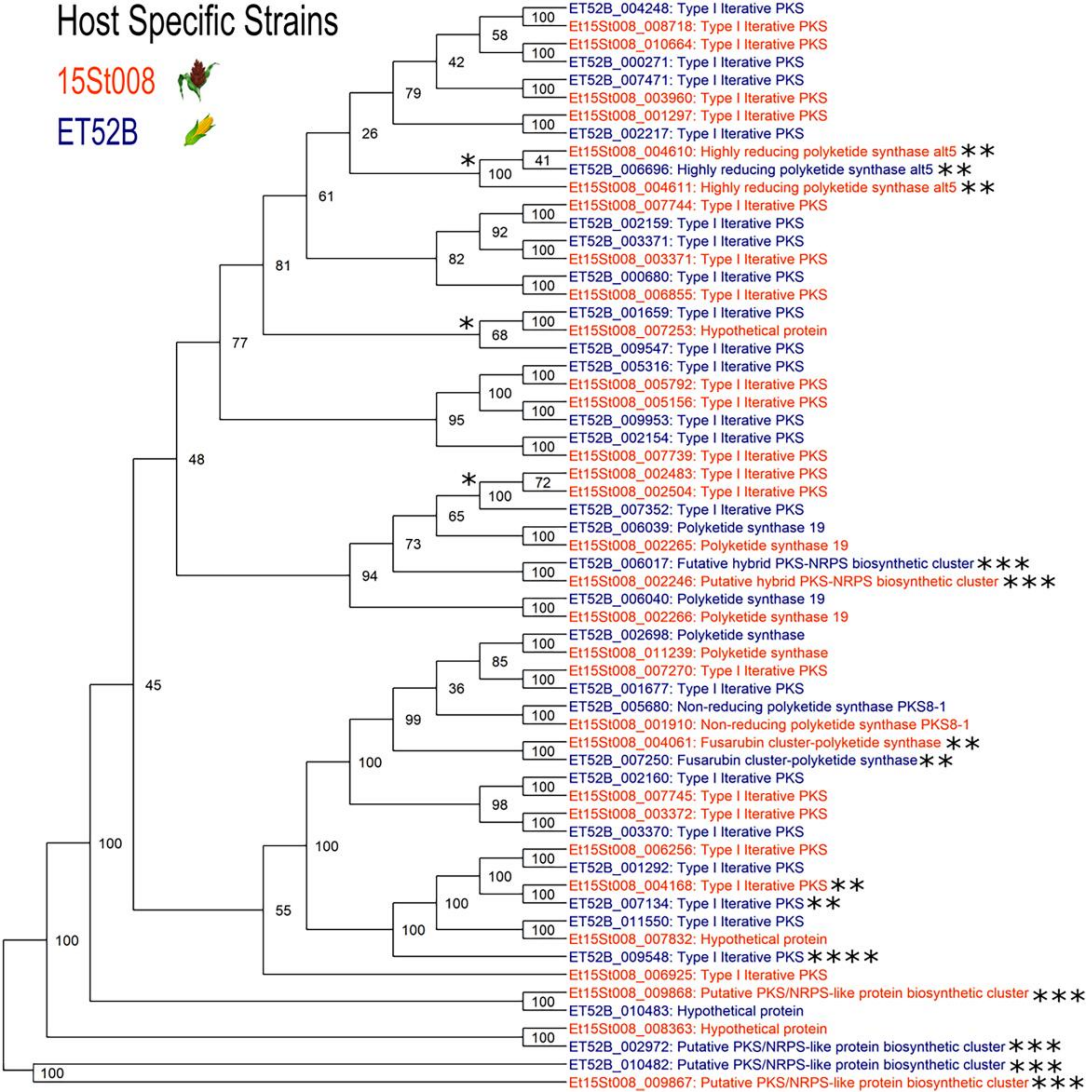
Phylogenetic analysis showing the relationship between the PKS encoding genes in the sorghum- and maize-specific strain genomes. The nodes that have a cluster of three genes have undergone a duplication event in one of the strains and are marked with a single asterisk. The genes on the contig associated with maize virulence (Et52B_contig10/15St008_contig7) are marked with a double asterisk. The hybrid genes are emphasized with three asterisks. The maize-specific gene that does not have a sorghum-specific homolog is marked with four asterisks.

**Fig. 3.**
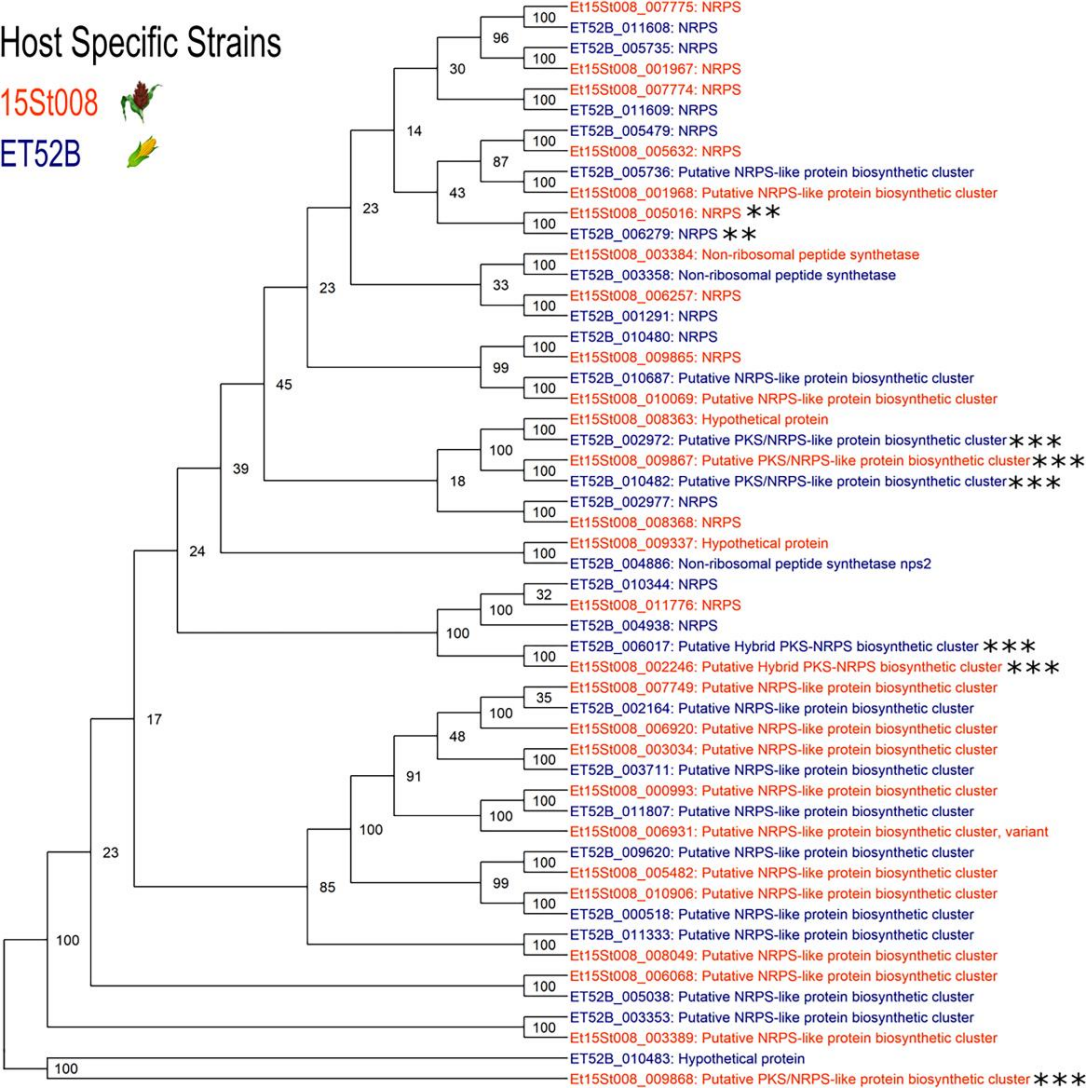
Phylogenetic analysis showing the relationship between the NRPS encoding genes in the sorghum- and maize-specific genomes. The nodes that have a cluster of three genes have undergone a duplication event in one of the strains and are marked with a single asterisk. The genes on the contig associated with maize virulence (Et52B_contig10/15St008_contig7) are marked with a double asterisk. The hybrid genes are emphasized with three asterisks.

The maize-specific strain had 26 PKS-encoding genes and 22 NRPS-encoding genes. The sorghum-specific strain had 25 PKS-encoding genes and 23 NRPS-encoding genes. Both strains had three PKS–NRPS hybrid encoding genes (Table 3). A phylogenetic analysis was conducted to analyze the relatedness within the PKS and NRPS-encoding gene groups. The majority of the PKS and NRPS-encoding genes had a single homolog in each host-specific strain (Fig. 2 and Fig. 3). There were two PKS-encoding genes in the maize-specific strain that each had two homologs in the sorghum-specific strain; and one PKS-encoding gene in the sorghum-specific strain that had two homologs in the maize-specific strains (Fig. 2).

**Table 3.** Comparison of the number of PKS, NRPS, and PKS–NRPS hybrid genes between the sorghum- (15St008) and maize-specific (Et52B) strains.

	Et52B	15St008
PKS genes		
Type I iterative PKS	20	18
Polyketide synthase 19	2	2
Polyketide synthase	1	1
Nonreducing polyketide synthase PKS8-1	1	1
Highly reducing polyketide synthase alt5	1	2
Fusarubin cluster-polyketide synthase	1	1
Total	26	25
NRPS genes		
Nonribosomal peptide synthetase	2	2
NRPS	10	9
Putative NRPS-like protein biosynthetic cluster	10	12
Total	22	23
PKS–NRPS hybrid genes		
Putative hybrids PKS–NRPS biosynthetic cluster	1	1
Putative PKS/NRPS-like protein biosynthetic cluster	2	2
Total	3	3

There was one maize-specific type I iterative PKS-encoding gene (Et52B_009548) that did not have a homolog in the sorghum-specific strain (Fig. 2). Two NRPS-encoding genes in the maize-specific strain each had two homologs in the sorghum-specific strain. One maize-specific NRPS-encoding gene (Et52B_004886) aligned with a sorghum-specific gene that was annotated as a hypothetical protein (Fig. 3). The PKS–NRPS hybrid encoding genes were included in both phylogenetic trees. There were the same number of hybrids in both strains, however, they did not all align with each other. Two of the hybrid genes had a homolog of a hypothetical protein (Fig. 2 and Fig. 3).

### Gene expression differences

The quality control analysis conducted with FastQC indicated that quality scores were above 35 for the RNA sequence reads for all samples, and no adaptors were present. In general, more reads from the axenic samples aligned to the fungal genomes than those from the *in planta* samples (Supplementary Table d). The unmapped reads that did not align with the fungal reference genomes in the *in planta* samples aligned to either the maize or sorghum genome and were thus not an indication of poor quality reads. Each axenic sample had ∼24–40 million assigned reads and each *in planta* sample had ∼1–5 million assigned reads (Table 4).

**Table 4.** Percentage of RNA reads and number of reads in millions assigned to a gene region across different samples. The 15St008 and SLB are sorghum-specific samples, and reads were assigned to the 15St008 genome. The Et52B and NLB are maize-specific samples, and reads were assigned to the Et52B genome.

Sample name	Culture type	% Assigned	No. assigned (M)
15St008_1	Axenic	77.6	30.5
15St008_2	Axenic	76.9	39.5
15St008_3	Axenic	78.2	28.5
SLB2	*In planta*	80.3	1.9
SLB4	*In planta*	79.0	2.2
SLB6	*In planta*	71.3	0.7
Et52B_1	Axenic	41.9	28.2
Et52B_2	Axenic	35.9	28.7
Et52B_3	Axenic	17.1	24.6
NLB1	*In planta*	69.0	4.6
NLB2	*In planta*	58.8	2.1
NLB5	*In planta*	62.4	5.2

The analysis pipeline identified 5,829 DEGs in the maize-specific strain (Supplementary Table e). A total of 2,719 genes had a positive fold change, and 3,110 genes had a negative fold change (positive fold change was upregulated *in planta*, and negative fold change was downregulated *in planta*). Of the DEGs in the maize-specific strain, 158 were unique to the maize-specific strain's genome, 1,826 were genes with homologs in the sorghum strain but only differentially expressed in the maize-specific strain, and 3,841 were genes with homologs in the sorghum strain that were differentially expressed in both strains (Supplementary Fig. b).

**Fig. 4.**
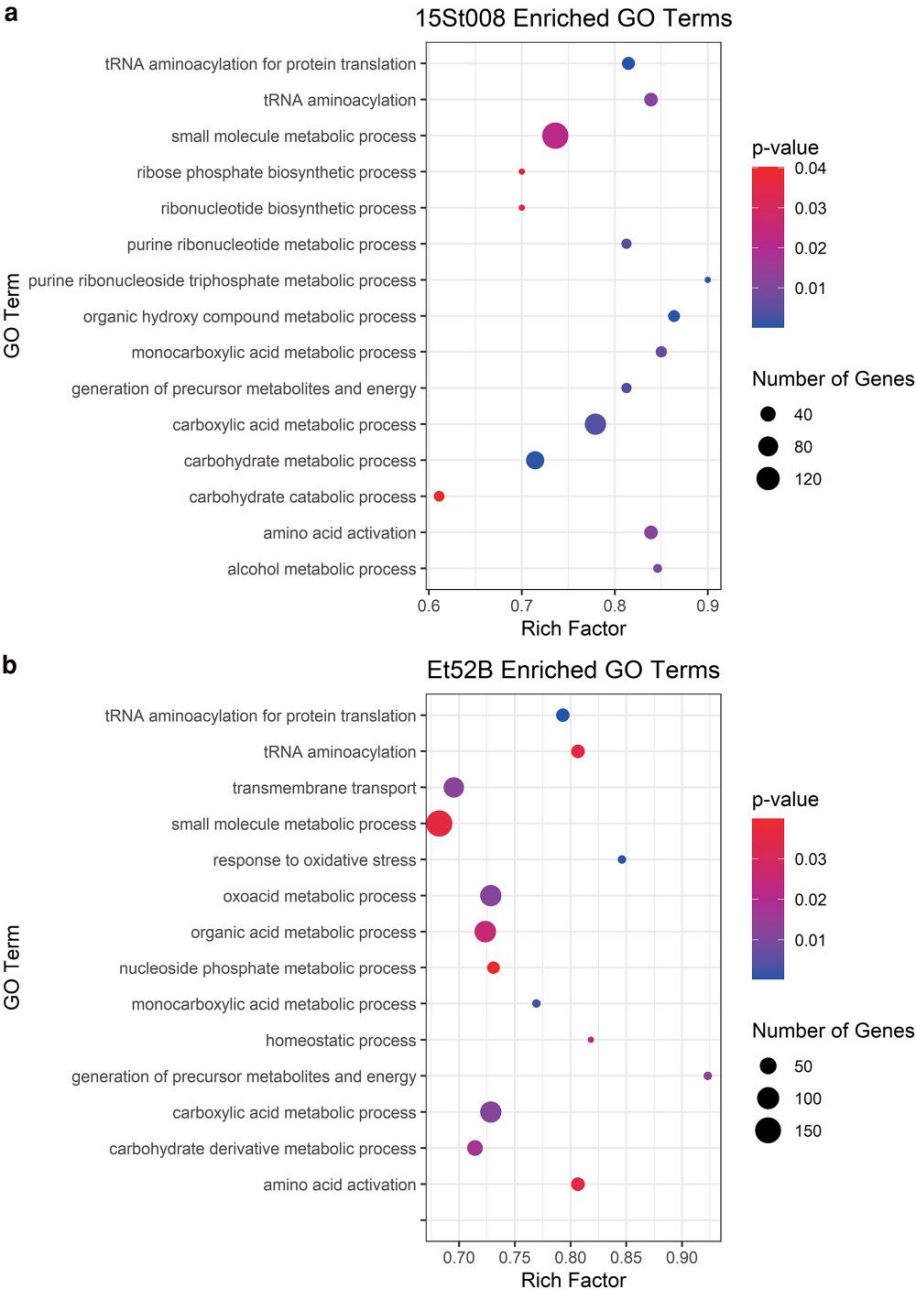
DEG *P*-values from the transcriptome data were used in a Kolmogorov–Smirnov test to determine enriched biological GO terms in the (a) sorghum-specific genome and the (b) maize-specific genome.

**Fig. 5.**
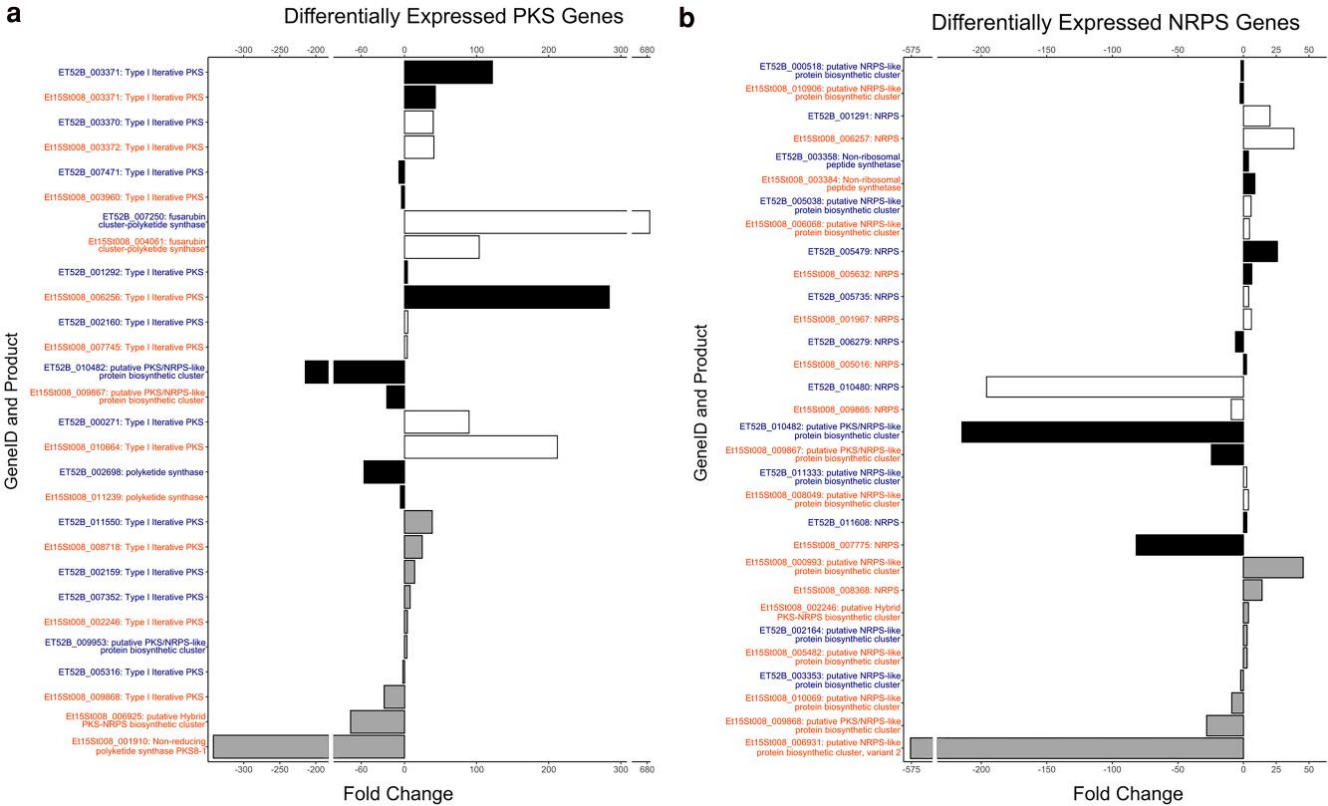
Bar plots of the significantly differentially expressed a) PKS genes and b) NRPS genes. The genes that had a positive fold change had increased expression in the *in planta* samples compared with the axenic samples. The genes with orange lettering are from the sorghum-specific strain, and the genes with blue lettering are from the maize-specific strain. The genes are grouped by homologs. The black and white bar colors represent genes that are homologs to each other. The gray bars are genes that either do not have a homolog in the other strain or the homolog was not significantly differentially expressed.

A total of 177 of the maize-specific strain predicted effectors were differentially expressed with 93 upregulated *in planta*. Two effectors (ET52B_002356 and ET52B_009117) were unique to the maize-specific strain. Fourteen biological process GO terms were enriched based on the DEG data in the maize-specific strain (Fig. 4b). There were 14 PKS genes differentially expressed in the maize-specific strain (Fig. 5a). Four of these were unique to the maize-specific strain, meaning that they did not have a homolog in the sorghum-specific strain. There were 13 NRPS genes differentially expressed in the maize-specific strain (Fig. 5b).

There were 5,580 DEGs in the sorghum-specific strain, with 2,449 genes having a positive fold change and 3,131 genes having a negative fold change (Supplementary Table e). Of the DEGs in the sorghum-specific strain, 148 were unique to the sorghum-specific strain's genome, 1,586 had a homolog in the maize-specific strain but were only differentially expressed in the sorghum-specific strain, and 3,842 had a homolog and were differentially expressed in both strains (Supplementary Fig. b). In both the maize- and sorghum-specific strains, four DEGs were not included in the homolog analysis because they were tRNA and did not have a transcript.

A total of 168 of the sorghum-specific strain effectors were differentially expressed with 102 upregulated *in planta.* Four effectors (Et15St008_001167, Et15St008_001166, Et15St008_002882, and Et15St008_006910) were unique to the sorghum-specific strain. Fifteen biological process GO terms were enriched based on the DEG data in the sorghum-specific strain (Fig. 4a). There were 14 PKS genes differentially expressed in the sorghum-specific strain (Fig. 5a). Four were unique to the sorghum-specific strain. There were 17 NRPS genes differentially expressed in the sorghum-specific strain (Fig. 5b). Five of these NRPS genes were differentially expressed in both strains, with two of them having an opposite expression pattern between the two strains. One of these two genes was on the contig associated with virulence to maize and was upregulated during infection in the sorghum-specific strain and downregulated during infection in the maize-specific strain (Fig. 5b).

### QTL location

We translated the positions of QTL identified using the biparental mapping population (Singh *et al*. 2022) onto our new reference assemblies. Virulence to maize localized to scaffold CP054627 of Et28A in Singh *et al* (2022) corresponded to contig 10 of the maize-specific strain Et52B in this study. There were 1,090 genes on the maize-specific strain's contig 10. Of the 57 genes unique to the maize-specific strain, three were PKS genes, one was an NRPS gene, and 50 were in CAZy families. The contig 10 of Et52B was syntenic to the contig 7 of the sorghum-specific strain (15St008) (Fig. 1a). There were 1,059 genes on contig 7 of the sorghum-specific strain. Of these genes, 32 were unique to the sorghum-specific strain, four were PKS-encoding genes, one was an NRPS-encoding gene, and 48 were in CAZy families. In addition, there are differences in gene content between the maize- and sorghum-specific strains. Thus, there is an additional PKS-encoding gene in the sorghum-specific strain and two additional genes in the CAZy families in the maize-specific strain. Singh *et al*. (2022) did not map any significant markers for virulence to sorghum.

### Candidate genes for host specificity

The genome annotations, effector predictions, genome comparisons, transcriptome data, and genetic mapping results were combined to identify candidate host-specificity genes (Supplementary Fig. a). For the maize specificity locus, the region of interest was located on contig 7 of the sorghum-strain genome and contig 10 of the maize-strain genome. In these genomic regions, there were three effectors all annotated as hypothetical proteins with increased expression during maize infection and very low expression during sorghum infection. One of these effectors (ET52B_007082/Et15St008_004220) has a deletion in the sorghum-specific strain causing it to lose its secretion signal. There were no differences in amino acid sequences in the other two effectors (ET52B_006580/Et15St008_004720 and ET52B_007221/Et15St008_004089) between the maize- and sorghum-specific homologs. No candidate genes were identified in these genomic regions that were expressed during sorghum infection but not in maize (Supplementary Fig. a).

For the sorghum-specificity locus, three potential regions of interest with translocations were identified (i) translocation between 15St008 contig5 and ET52B_contig11, (ii) translocation between 15St008_contig12 and ET52B_contig6, and (iii) translocation between 15St008 contig 14 and ET52B contig 7 (Supplementary Fig. a). The translocation between 15St008_contig5 and ET52B_contig11 along with a small inversion contained a type I iterative PKS gene (ET52B_007352) that had increased expression during maize infection. This gene was duplicated in the sorghum-specific strain (Et15St008_002483 and Et15St008_002504) and had no detectible expression across all the sorghum infected samples. This PKS gene could potentially be involved in maize infection but act as an avirulence gene to sorghum infection. The translocation between 15St008_contig12 and ET52B_contig6 contained two genes in the sorghum-specific strain (Et15St008_008491 and Et15St008_008688) that were classified as glycoside hydrolase family by InterPro. These two genes had high levels of expression across all *in planta* samples and axenic samples. The homologs of these genes (ET52B_003106 and ET52B_003306) had a negative fold change in the maize-specific strain, meaning they are down regulated during maize infection. There was another gene (Et15St008_008707) in the sorghum-specific strain that was also classified into a glycoside hydrolase family that had a positive fold change, meaning that was upregulated during sorghum infection. The homolog of this gene (ET52B_003326) had very low levels of expression in the maize-specific strain across all *in planta* and axenic samples. There were no candidate genes in the translocation between contig 14 of the sorghum-specific strain and contig 7 of the maize-specific strain.

## Discussion

Understanding the genetic factors involved in pathogen host specificity can potentially be exploited to develop resistant cultivars. The genes underlying host specificity in *E. turcicum* are unknown. To investigate host specificity in this pathosystem, we compared the genomes and transcriptional responses of a maize- and a sorghum-specific *E. turcicum* strain. A whole-genome comparison identified 10 inversions, three translocations, and homologs between the two strains and gene duplication events within each strain. The whole-genome assembly also enabled us to reanalyze genetic mapping data and examine gene expression profiles to identify potential candidate genes associated with host specificity.

In this comparative genome study, we assembled and annotated both the sorghum-specific and the maize-specific *E. turcicum* strains’ genomes using the same methods. Previous genomic comparisons between strains with differing host specificities utilized genomes assembled and annotated using different methods (Ma *et al*. 2022), that likely influenced the results of the comparative analysis. This is apparent in both our synteny and the ortholog analysis that compared our genome sequences to Et28A-v2.0. There were more genomic and structural differences between the two maize-specific strains Et52B and Et28A-v2.0 than there were between our sorghum and maize-specific strain (Fig. 1). We believe this was due to differences in assembly, annotation, and sequencing methods. The *E. turcicum* genome assembled by Ma *et al*. (2022) for the sorghum-specific strain GD003 had a lower N50 value (11,965 bp) compared with our two genomes, and a BUSCO analysis was not reported. The GD003 assembly had 22 contigs and a total of 10,428 protein-coding genes (Ma *et al*. 2022). The Et28A-v2.0 genome was assembled into scaffolds with a scaffold N50 of 8 Mb. Et28A-v2.0 had 8,276 protein-coding genes (Ohm *et al*. 2012; Condon *et al*. 2013).

Structural variations, such as translocations and inversions, are known to affect gene expression (Hartmann 2022), and thereby altering phenotypes. Four inversions and two translocations were common between the sorghum- and maize-specific genomes (ET52B) examined in this study and between the sorghum-specific strain and the maize-specific genome Et28A-v2.0, indicating that there may be consistent structural differences between the two *E. turcicum formae speciales*. However, our study only included one sorghum-specific *E. turcicum* strain. A high level of genetic diversity has been reported within both *E. turcicum* f. sp. *zeae* and *E. turcicum* f. sp. *sorghi* in multiple regions of China (Tang *et al*. 2015). In studies focusing on *E. turcicum* strains from sorghum, genetic diversity varied among regions across China, with moderate levels of biological genetic diversity (Cui *et al*. 2022). *E. turcicum* isolates sampled from two locations in South Africa exhibited moderate levels of genetic diversity with some regional variation (Nieuwoudt *et al*. 2018). These results suggest that structural variation between the *formae speciales* may be a variable trait thus requiring comparative genomics with additional strains. However, based on the data we have, one of the consistent inversions is located on the contig associated with maize virulence, which could potentially affect the locus involved in maize infection or its expression. Such structural variants are also implicated in regions enriched with pathogenicity factors, playing a role in the rapid adaptation of pathogens including host specialization (Hartmann 2022). This is evident in *C. heterostrophus*, where a large translocation affected the expression of toxin encoding genes (Haridas *et al*. 2023).

Previous studies have shown that separate loci are responsible for virulence in maize and sorghum, and the underlying mechanism causing host specificity is most likely qualitative (Hamid and Aragaki 1975; Singh *et al*. 2022). Once the host specificity in *E. turcicum* led to the separate *formae speciales,* it would be expected that the two host-specific strains would continue to adapt to their respective hosts. This adaptation over time could explain the additional syntenic differences observed between the two strains and could be reflected in the genetic divergence reported between maize and sorghum strain in previous research (Cui *et al*. 2023). Furthermore, several differences in the PKS and NRPS gene groups between the strains could also be due to this host-driven adaptation. There is evidence of multiple gene duplication events along with expression variations in both strains for PKS and NRPS genes. Two maize-specific PKS genes (Et52B_011550 and Et52B_001659) aligned to hypothetical proteins in the sorghum-specific strain that had large deletions, which likely prevented them from functioning as PKS genes in sorghum-specific strain. This continual adaptation to specific host could also explain the differences observed in the enriched biological GO terms, suggesting uniquely enriched GO terms in the host-specific strains are critical for infection of each host. We previously reported a range in aggressiveness for host-specific isolates, and the isolates able to infect both maize and sorghum were overall less aggressive in a screen of a biparental population (Singh *et al*. 2022). This could be due to offspring of the two *formae speciales* inheriting the locus for host specificity but lacking additional genes required for enhancing infections to the specific host, leading to virulent but less aggressive isolates.

The importance of effectors in host specificity is reported in several pathogens (Li *et al*. 2020). On the contig associated with maize virulence (Et52B_contig10), there are three effectors potentially acting as virulence factors to maize and could be related to host specificity. Each of these effectors has high levels of expression during maize infection and low expression in all the sorghum-specific strain samples. The inversion seen between Et52B_contig10 and 15St008_contig7 could have caused the loss of expression of these effectors in the sorghum-specific strain. One of these effectors (ET52B_007082/Et15St008_004220) is of special interest because it has a deletion in the sorghum-specific strain causing it to lose its secretion signal. The loss of function of a virulence gene necessary for maize infection could result in a sorghum-specific strain.

Effectors and secondary metabolites are reported to play a role in host specificity in pathogens including *M. oryzae* (Bohnert *et al*. 2004; Inoue 2017). In our study, we identified a type I iterative PKS gene as a potential candidate involved in host specificity. In the maize-specific strain, this gene (ET52B_007352) had increased expression for *in planta* samples. This gene was duplicated in the sorghum-specific strain (Et15St008_002483 and Et15St008_002504) and both genes had undetectable expression across all the replicates of the transcriptome evaluation. This PKS-encoding gene could be an important pathogenicity factor in maize but in sorghum, recognized by resistance proteins, it triggers an immune response, thus functioning as an *AVR* gene. The amino acid sequence encoded by this type I iterative PKS gene was identical between the maize-specific strain and the sorghum-specific strain. It is possible that the expression of this gene was disrupted during the duplication event in the sorghum-specific strain. The loss of function of this gene could have enabled *E. turcicum* to cause disease on sorghum. This PKS gene is located on 15St008_contig5 and Et52B_contig11 in the translocation between these two contigs. The location of this gene could explain why sorghum virulence could not be genetically mapped in the biparental population.

We identified multiple candidate genes potentially involved in host specificity in the translocation region between 15St008_contig12 and Et52B_contig6 that could contain the virulence to sorghum locus. The most significant QTL marker for virulence to sorghum that Singh *et al* (2022) mapped using a different reference genome aligned to 15St008_contig12. The three genes (Et15St008_008707, Et15St008_008491, and Et15St008_008688) in this translocation classified as glycoside hydrolase families could be part of a biosynthetic cluster that synthesizes a secondary metabolite required for sorghum infection. The homologs of these three genes (ET52B_003326, ET52B_003106, and ET52B_003306) had low expression during maize infection. If these three glycoside hydrolase genes in the putative biosynthetic cluster were not expressed in the maize-specific strain during *in planta* infection that could prevent the synthesis of the secondary metabolite required for sorghum infection. There are several other genes in this translocation that were classified into InterPro domains related to secondary metabolites that could be part of the putative biosynthetic gene cluster. These genes have significant levels of expression during *in planta* infection in both strains. Of these genes, four were in glycoside hydrolase families, two were in mycotoxin biosynthesis protein families, and three were in PKS families.

In a previous genomic comparison study, Ma *et al*. (2022) identified an effector protein-encoding gene StCEL2 (Et28A-v2.0_A2464), which encodes an endo-1,4-B-D-glucanase, as playing an important role in maize infection. This effector was upregulated during maize infection and was annotated as a glycoside hydrolase family 7 protein with endo-1-4-B-D-glucanohydrolase activity, an important component of cellulase genes (Ma *et al*. 2022). Cellulase activity has been reported to be higher in maize-specific strains than sorghum-specific strains (Tang *et al*. 2015). Ma *et al*. (2022) speculated that this difference in cellulase activity could be an underlying mechanism of host specificity. While the increase in cellulase activity could represent an adaptation of the maize-specific strain to its host, it may not be necessary for maize infection. In our study, the ortholog of this gene was found in both *E. turcicum* genomes (15St008 and Et52B); however, it had low expression in both strains across all axenic and planta samples. Proper identification of the genes involved in host specificity will require gene deletions, complementation, and allele swapping between maize- and sorghum-specific strains.

### Conclusion

Two high-quality whole-genome assemblies of a maize- and a sorghum-specific *E. turcicum* strain were created, providing valuable resources for the continued research on host specificity in this pathosystem. Our study confirmed that there are large-scale structural variants between host-specific strains of *E. turcicum*. We explored the potential role of effectors and secondary metabolites in host specificity and identified key differences in putative effectors, PKS-encoding genes, and NRPS-encoding genes between the two strains. Using genetic mapping of a biparental population previously generated between the two sequenced strains, we identified a contig associated with virulence to maize. Additionally, combining mapping data with the transcriptional data enabled identification of candidate host-specificity genes. These findings advance our understanding of genetic factors contributing host specificity and pathogenicity in *E. turcicum*, offering potential targets for future research and development of more effective resistance strategies.

## Supplementary Material

jkaf084_Supplementary_Data

## Data Availability

The genome and transcriptome sequences along with the annotations have been deposited in NCBI BioProjects: PRJNA1187605 and PRJNA1187604. Transcriptome accessions: GSE282476 and GSE282460. Genome assembly and annotation accessions: JBMGSY000000000 and JBMGSX000000000. Supplemental material available at G3 online.
