# Supplementary material for: Identification of candidate host-specificity genes in Exserohilum turcicum using comparative genomics and transcriptomics
Source: G3 (Bethesda). 2025 Apr 11;15(6):jkaf084. doi: 10.1093/g3journal/jkaf084 (PMC12135012; doi:10.1093/g3journal/jkaf084)
Supplement: jkaf084_Supplementary_Data [file jkaf084_supplementary_data.zip › File_S1_G3-2025-405655.pdf]

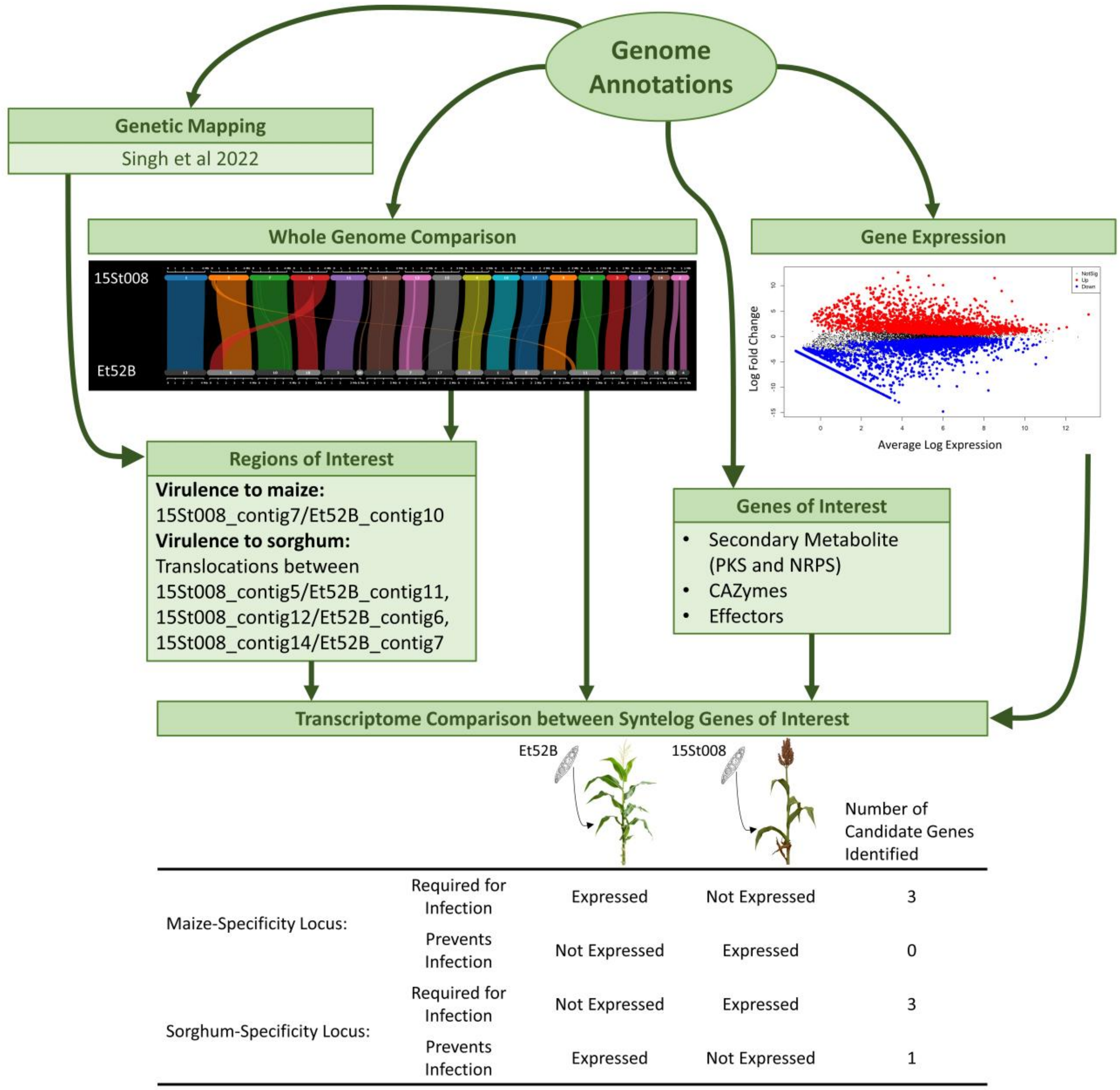

Figure A: Pipeline showing how different data sets and experiments were used to identify candidate host-specificity genes in *Exserohilum turcicum*. The annotations of the maize-specific (Et52B) and sorghum-specific (15St008) genomes were used to complete a whole genome comparison between the two strains. Transcriptome data and the genome annotations were used to identify differentially expressed genes and their fold change. Previous genetic mapping and synteny plots were used to find the regions suspected to contain the maize virulence and sorghum virulence loci. Expression data was compared between the strains during *in planta* infection to identify candidate genes.

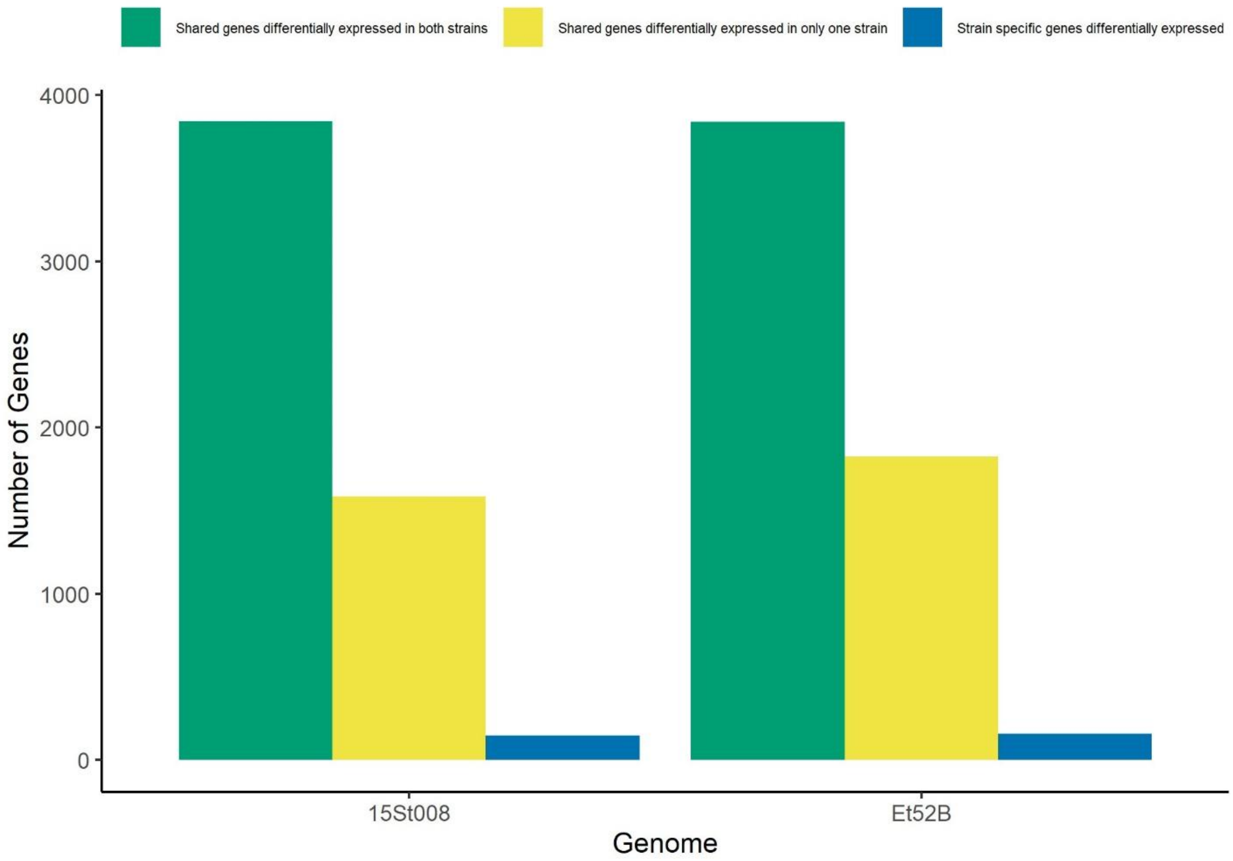

Figure B: Syntelog comparison of the differentially expressed genes in the sorghum- and maize-specific genomes.
